# Examining Inequities in Incidence of Catastrophic Health Expenditures on Different Healthcare Services and Health Facilities in Nigeria

**DOI:** 10.1371/journal.pone.0040811

**Published:** 2012-07-16

**Authors:** Obinna Onwujekwe, Kara Hanson, Benjamin Uzochukwu

**Affiliations:** 1 Health Policy Research Group, Department of Pharmacology and Therapeutics, University of Nigeria Enugu-Campus, Enugu, Nigeria; 2 Department of Health Administration and Management, University of Nigeria Enugu-Campus, Enugu, Nigeria; 3 Department of Global Health and Development, London School of Hygiene and Tropical Medicine, London, United Kingdom; 4 Department of Community Medicine, College of Medicine, University of Nigeria Enugu-Campus, Enugu, Nigeria; Johns Hopkins University, United States of America

## Abstract

**Objective:**

There is limited evidence about levels of socio-economic and other differences in catastrophic health spending in Nigeria and in many sub-Saharan African countries. The study estimated the level of catastrophic healthcare expenditures for different healthcare services and facilities and their distribution across socioeconomic status (SES) groups.

**Methods:**

The study took place in four Local Government Areas in southeast Nigeria. Data were collected using interviewer-administered questionnaires administered to 4873 households. Catastrophic health expenditures (CHE) were measured using a threshold of 40% of monthly non-food expenditure. We examined both total monthly health expenditure and disaggregated expenditure by source and type of care.

**Results:**

The average total household health expenditure per month was 2354 Naira ($19.6). For outpatient services, average monthly expenditure was 1809 Naira ($15.1), whilst for inpatient services it was 610 Naira ($5.1). Higher health expenditures were incurred by urban residents and the better-off SES groups. Overall, 27% of households incurred CHE, higher for poorer socioeconomic groups and for rural residents. Only 1.0% of households had a member that was enrolled in a health insurance scheme.

**Conclusion:**

The worse-off households (the poorest SES and rural dwellers) experienced the highest burden of health expenditure. There was almost a complete lack of financial risk protection. Health reform mechanisms are needed to ensure universal coverage with financial risk protection mechanisms.

## Introduction

Health expenditures are said to be “catastrophic” when they risk sending a household into, or further into, poverty [1]. This is usually measured by setting a reference or standard, and counting the number of households for whom their level of health expenditure in a given period can be said to be catastrophic. Nigerians are particularly at risk of incurring catastrophic health expenditures (CHE) because of the high level of prevalent user fees and predominant use of out-of-pocket spending (OOPS) to pay for health expenditures in the health system. Catastrophic health expenditures usually result from high levels of out-of-pocket spending (OOPS) on healthcare by households, resulting from user fees for the services.

Health care financing systems which depend on user fees, defined as payments made at the point of service use and there is no risk sharing [2] are particularly likely to generate catastrophic levels of health expenditure. User fees are mostly paid as out-of-pocket spending (OOPS) in Nigeria and in many sub-Saharan African (SSA) countries because of lack of financial risk pooling mechanisms [3].

Nigeria introduced user fees for government health services within the framework of the Bamako Initiative revolving drug funds [4], [5]. As in many SSA countries in the 1980s, the introduction of user fees in Nigeria was arguably in response to the severe difficulties in financing health services. Despite commitments to increase the share of government expenditure that is devoted to health [6], private expenditure on health has remained very high in Nigeria resulting from user fees for health services.

In Nigeria, households and firms have been shouldering around 70% of total health expenditure and 90% of these private expenditures are non-pooled as most of it takes place via OOPS [7], [8], [9]. A number of studies have found some evidence of catastrophic health expenditures (CHE) arising from spending on specific health conditions in Nigeria, including OPD management for heart failure, drugs for HIV and treatment of malaria [10], [11], [12]. Excessive health expenditures have also been demonstrated to lead to impoverishment. It was found that impoverishment in India due to OOPS was highest in people living below the poverty line compared to people above the line [13].

The incidence of CHE is enhanced by the lack of pooling schemes for health financing [14]. The level of catastrophic payments increases as the volume of total health expenditure met by out-of-pocket payments increases [15]. With 70% of the population living below the $1-a-day poverty line in Nigeria [16], payments for health services in the form of user charges are likely to present a barrier to access [2]. A survey in Nigeria shows that non-availability of cash at time was the major constraint to accessing healthcare services [17]. User fees paid through OOPS also exacerbates already inequitable access to quality care [18].

Expanded access to risk-pooling financing mechanisms such as health insurance is an important route to better financial risk protection and decreased incidence of CHE. The Commission on Macroeconomics and Health recommends that OOPS should be channelled into community risk-pooling financing schemes to help cover the costs of community-based health delivery [19]. It was found that with removal of OOPS for medicines or out-patient department visits (OPD), the share of households falling into poverty decreased from 5% to 0.5% [13]. At present there are two main risk-pooling mechanisms in Nigeria: the National Health Insurance Scheme (NHIS) and community-based health insurance (CBHI). Currently, the NHIS only covers federal government employees and the coverage level is less than 5% of the general population [20]. CBHI schemes are scattered and cover only small numbers of households, though the National Health Insurance Council (NHIC) plans to promote and scale-up community-based health insurance (CBHI) schemes in the country.

The paper provides new knowledge about inequities in the incidence of CHE arising from different healthcare services and facilities in Nigeria. There is little existing information about socio-economic and other differences in the incidence of catastrophic spending especially the contribution of expenditures in public and private facilities to overall incidence of CHE. Existing literature has mostly focused on CHE due to total health expenditure, but has not specifically examined CHE arising from expenditures on different providers and different services, and the impoverishing consequences of use of inpatient and outpatient services. The findings may contribute to improved financing and provision of healthcare services in Nigeria, so as to increase demand for services without the attendant catastrophe and possible impoverishment.

## Methods

The study was approved by the University of Nigeria Teaching Hospital Ethics Committee. All respondents gave written informed consent.

### Research Area

The research was undertaken in 4 selected Local Government Areas (LGAs) in two states in south eastern Nigeria. One rural and 1 urban LGA were selected from Enugu and Anambra states, respectively (2 LGAs per state), so as to enable a comparison of incidence of catastrophic health expenditures (CHE) between the urban and rural areas. The two state capitals were selected as the urban LGAs. The two rural LGAs were purposively selected. Enugu is the capital city of Enugu state, which has an estimated population of about 3,100,000. Anambra state has a population of 4,054,824 and its capital city is Awka. Each state capital has a tertiary hospital and each urban LGA has a public general hospital. There are health centres in all rural LGAs. The private sector is represented by a diverse set of health care providers including private hospitals, clinics, pharmacies, patent medicine dealers (PMDs) and mission hospitals, all of which are found in both states.

### Data Collection

The sample size per state was determined based on a power of 80% and 95% confidence level to detect statistical differences in key variables. The minimum sample size was 1166 per LGA. However, the sample size was increased to at least 1200 per LGA so as to account for refusals. A pre-tested questionnaire was administered by trained field workers to at least 4,800 randomly selected householders from 4 LGAs In each selected household, one woman (the primary care giver) – or in her absence the male head of the household was interviewed.

The questionnaire was used to collect information on household healthcare and other expenditures. Data were collected on the type of provider where the expenditures were incurred – whether public or private facilities and on the type of facility. Health expenditures were also broken down by whether they were for outpatient or inpatient care. A one month recall period was used to collect data on health expenditures for outpatient visits so as to reduce the incidence of recall bias that would occur if longer periods were used. A six-month recall period was used for in-patient admissions and expenditure, because such events are rarer than out-patient visits. The questionnaire also collected data on household expenditure on fuel, rent, school fees, leisure, clothing and food.

### Data Analysis

Several thresholds for measuring CHE have been proposed by different researchers in different settings. Some authors used a threshold of 40% of “capacity to pay” which was defined as income after subsistence needs are met, which in practice amounts to income minus food expenditure [15]. Other authors used a threshold of 10% of total expenditure [21], [22], [23], [24]. Castillo-Riquelme et al. and Materia et al. presented their results using thresholds of 10% of household income and 40% of non-food income [25], [26]. For the poorest households, especially already living below the poverty line, any level of health expenditure can be catastrophic [11]. At this level of poverty, households may not have money to spend on any household need aside from food so some have argued that the threshold for assuming catastrophe may be less than 2% [1], [12]. Some authors advocate for the use of a variable threshold which is lower for lower socioeconomic groups and higher for those in higher groups [27].

For this study we used the conventional threshold of 40% of non-food expenditure in order for the results to be comparable to the international literature. In addition, we computed indices of catastrophe separately for inpatient and outpatient services, and for use of public and private providers. This allowed us to examine the extent to which different types of expenditure are responsible for financial catastrophe, and whether use of public services – intended to provide a safety net – is associated with catastrophic expenditures. The overall incidence of CHE was also computed using the 5% non-food expenditure threshold level.

For equity analysis, an urban–rural distinction and a socio-economic status (SES) index were used to examine the systematic differences in catastrophic costs. Principal components analysis (PCA) was used to create a SES index [28] using information on the households’ ownership of a: radio; bicycle; motorcycle; car; refrigerator; generator; kerosene lamp; together with the weekly household cost of food. The first principal component of the PCA was used to derive weights for the SES index. The highest weight was given to ownership of a fridge (0.53), followed by ownership of a television (0.50), ownership of a car (0.41), ownership of a generator (0.39), ownership of a radio (0.28), per capita food value (0.20), ownership of a bicycle (−0.15), ownership of a motorcycle (0.08), and ownership of a kerosene lamp (−0.03). The index was used to divide the households into five equal sized SES groups (quintiles). The quintiles were Q1 (most poor); Q2 (very poor); Q3 (poor); Q4 (less poor); and Q5 (least poor).

The frequency distributions of the variables by SES and rural-urban location were calculated and chi-squared (Chi^2^) tests for trend analysis were used to examine statistical differences across socioeconomic groups. The Kruskal-Wallis non-parametric test, a non-parametric equivalent of ANOVA, which also reports a Chi^2^ statistic, was used to compare differences in means of continuous variables.

## Results

### Socio-economic and Demographic Characteristics

There were 2,390 rural households and 2,483 urban households in the full sample. The overall average household size was 4.5 people. The mean age of the respondents was 41.6 years. The majority of the respondents were female (mostly wives) and had some formal education. Household weekly mean food expenditure was 3,143 Naira ($26.2). Annual household mean non-food expenditure was 95,029 Naira ($791.9). Most of the households owned functional radios and kerosene lamps. Bicycles, motorcycles, cars, and generators were the least commonly owned household assets.

### Expenditures on Healthcare Seeking

The mean monthly household health expenditure was US$19.6, of which expenditure in public health facilities was US$5.5. (Table 1) The remaining expenditure was incurred in the private sector. The average monthly household expenditure on outpatient care was US$15.1and for inpatient care was US$5.1.

**Table 1:** Average monthly total household expenditures on outpatient and inpatient care and expenditure in public health facilities.

	Mean (SD)	US$
Expenditure in public facilities	661.3 (3,445.7)	5.5
Total expenditure – outpatient care (public and private)	1,809.0 (4,612.0)	15.1
Total expenditure – inpatient care (public and private)	609.6 (4,249.1)	5.1
Expenditure on outpatient care in public facilities	457.8 (2,115.5)	3.8
Expenditure on inpatient care in public facilities	203.5 (2,725.9)	1.7
Expenditure on outpatient care in public hospital	422.7 (2,022.2)	3.5
Expenditure on outpatient care in primary healthcare (PHC) centre	48.3 (724.7)	0.4
Expenditure on inpatient care in public hospital	229.6 (3,233.0)	1.9
Expenditure on inpatient care in PHC centre	4.6 (144.1)	0.04

Out of 3187 instances of healthcare payments recorded in the survey, out-of-pocket spending (OOPS) was the predominant payment mechanism and it was used by 3150 (98.8%) of the people that had to make healthcare payments. Only one person claimed to have used private voluntary health insurance. Nine people claimed that they were able to get their expenditures reimbursed (usually by an employer), 9 paid by instalment and 4 paid in-kind.

Table 2 shows how spending on the different providers differed by different population groups. Urban residents spent more money than rural residents on public and private hospitals, pharmacy shops and laboratories. The table also shows that as SES increases, the expenditures on public and private hospitals, pharmacy shops, laboratories and home treatment increases. Conversely, as SES decreases, expenditures on PHC centers, PMDs and herbalists increases.

**Table 2:** Mean monthly household expenditures on treatment paid to different providers (Naira).

	Home	Privatehospital	Public Hospital	PHCcentre	PMD	Pharmacyshop	Herbalist	LAB	Others
Mean expenditures on treatment by urban-rural residence									
Urban	18.7 (467.1)	1320.8 (6357.4)	720.8 (3595.1)	24.5 (298.1)	231.9 (934.5)	283.1 (1031.8)	2.0 (48.3)	60.4 (442.9)	207.9 (5424.1)
Rural	1.5 (44.6)	624.3 (3776.2)	427.1 (4970.4)	46.1 (450.4)	300.2 (1949.0)	78.1 (1193.9)	3.2 (112.6)	23.6 (441.8)	260.8 (3233.9)
X2 (p-value)	1.4 (.23)	68.1 (.0001)	98.4 (.0001)	3.0 (.082)	12.7 (.0001)	435.4 (.0001)	0.43 (.51)	48.4 (.0001)	5.3 (.022)
Mean expenditures on treatment by SES									
Quintile 1	2.3 (64.5)	380.5 (1766.5)	161.9 (1194.4)	53.8 (582.7)	340.6 (2668.9)	99.0 (1142.3)	5.6 (172.1)	44.0 (661.5)	263.2 (3641.2)
Quintile 2	4.4 (120.8)	721.7 (4411.1)	417.8 (3338.5)	54.5 (468.1)	285.0 (1020.8)	120.7 (1376.8)	2.4 (42.7)	19.7 (297.9)	281.0 (2718.9)
Quintile 3	1.0 (25.0)	903.2 (3797.3)	608.3 (3731.4)	19.4 (186.9)	269.2 (1359.7)	180.9 (700.1)	1.1 (24.6)	45.9 (375.7)	481.4 (8821.6)
Quintile 4	32.1 (698.9)	1172.1 (4748.1)	926.2 (6521.5)	28.0 (270.7)	264.7 (1076.7)	243.0 (956.1)	1.5 (45.0)	31.1 (259.7)	82.9 (1196.6)
Quintile 5	11.5 (232.4)	1726.4 (8854.9)	774.2 (4929.5)	19.4 (233.4)	166.4 (576.7)	271.5 (1277.9)	2.4 (53.9)	71.6 (494.3)	59.3 (893.3)
X2 (p-value)	1.2 (.88)	75.8 (.0001)	105.3 (.0001)	3.6 (.47)	52.9 (.0001)	177.0 (.0001)	3.8 (.44)	35.2 (.0001)	19.0 (.0001)
Concentrationindex	0.36	0.26	0.24	−0.22	−0.11	0.20	−0.25	0.13	−0.21
**Total**	10.3 (335.1)	979.8 (5265.7)	577.2 (4324.6)	35.1 (380.4)	265.3 (1518.2)	182.8 (118.6)	2.6 (86.0)	42.4 (442.7)	233.8 (4488.6)

### Incidence of Catastrophic Health Expenditure

The results show that 27% of households incurred monthly healthcare payments in excess of 40% of non-food expenditure (Table 3). Expenditure on out-patient care in public facilities led to 8% incidence of catastrophe at >40% threshold, whilst expenditures on out-patient care in all facilities (public and private) led 22% of households incurring a catastrophic level of expenditure. Expenditures on in-patient care in all facilities led to 3% incidence of CHE.

**Table 3:** Incidence of catastrophic expenditure for different services.

	Level of CHE
Monthly household total health expenditure	27%
Monthly household out-patient care expenditure in public facilities	8%
Monthly household out-patient care expenditure in all facilities	22%
Monthly household in-patient care expenditure in public facilities	1%
Monthly household in-patient care expenditure in all facilities	3%

Note: The incidence of catastrophic payments was 57% at a threshold of 5%.

Table 4 shows that the incidence of catastrophic health expenditures was generally greater in the rural areas compared to the urban areas. Incidence of catastrophic monthly total household expenditure (THE) increased as SES decreased (p<0.01). The table shows that for public sector out-patient care, the middle SES groups had highest incidence of CHE, whilst the most-poor SES had lowest level of CHE. However, for out-patient care in all facilities, CHE increased as SES decreased.

**Table 4:** Number of households from different population groupings with catastrophic health expenditures for different services and providers.

	CHE for total health expenditure (THE)n(%)	CHE for publicout-patient caren(%)	CHE for allout-patientcare n (%)	CHE for publicin-patient caren(%)	CHE for all in-patient caren (%)
Urban	378 (15%)	112 (5%)	350 (14%)	24 (1.0% )	77 (3%)
Rural	921 (39%)	132 (62%)	703 (30%)	25 (1.1% )	100 (4%)
X2 (p-value)	342.2 (.0001)	3.9 (.028)	179.5 (.0001)	.08 (.44)	5.3 (.013)
Quintile 1	383(40%)	29 (3%)	286(31%)	4 (0.4%)	29 (3%)
Quintile 2	302(31%)	47 (5%)	242(26%)	11 (1.2%)	30 (3%)
Quintile 3	248(26%)	65 (7%)	208(22%)	13 (1.4%)	40 (4%)
Quintile 4	184(19%)	64 (7%)	184(19%)	14 (1.5%)	38 (4%)
Quintile 5	181(18.6%)	39 (4%)	132(14%)	7 (0.7%)	40 (5%)
X2 (p-value)	155.7 (.00001)	19.6 (.001)	89.9 (.0001)	7.2 (.12)	2.7 (.61)
CI	−0.16	0.06	−0.15	0.03	0.06

### Use of Health Insurance as a Financial Risk Protection Mechanism

There were very low enrolment rates in health insurance and only 51 (1.0%) households had a resident who was a primary enrolee in a health insurance scheme (results not shown). The primary enrolees were mostly adults, and the formal sector programme of the NHIS was by far the most important insurance scheme. The number of people that were covered by the health insurance schemes was also very low.

## Discussion

The high incidence of catastrophic costs in the study area is worrying because over a quarter of households experienced levels of health expenditure that exceeded 40% of their non-food expenditure. In the lowest socioeconomic quintile, 40% of households incurred expenditure exceeding the conventional threshold for financial catastrophe. Financial catastrophe was higher in the rural areas where disposable income is lower.

All three of the key preconditions for catastrophic payments identified by Xu et al. [15] were found in this study; the availability of health services requiring payment, low capacity to pay, and the lack of prepayment or health insurance. Services are available, but there is a high level of private sector use, which always requires payment. Importantly, public sector facilities also charge for services, and therefore fail to perform their role as a social safety net for the poor. Poverty is high in Nigeria, with 70% of the population living below the $1 per day poverty line [16]. People paid mostly through out-of-pocket expenditure, with almost no health insurance, or other pre-payment or assured reimbursement payment mechanisms [12].

Previous data from Nigeria showed that on average, about 4% of households are estimated to spend more than half of their total household expenditures on healthcare and 12% of them are estimated to spend more than a quarter [9], [29]. Large differences were detected in the total burden of health expenditures both across socio-economic quintiles and geographic zones [29]. It was found that the mean percentage of income spent on total care for out-patient management of chronic heart disease for children was 16.3% in Nigeria [10]. It was found that the incidence of catastrophic health expenditures (CHE) in Nigeria was about 29% when a threshold of 5% of non-food expenditure was used [30]. Studies elsewhere have found various levels of CHE. Some authors showed that CHE was 5% in India [13]. CHE for cardiovascular disease hospitalisation was present in more than 50% of respondents in China, India and Tanzania [31].

It was insightful to find out that there were still high levels of CHE in the public sector, although the use of the private sector contributed more to incidence of CHE than the public sector. Nonetheless, the finding of CHE in the public sector is a possible indicator of the failing role of this sector in providing protection against CHE. Public healthcare should be the source of last resort especially for poor people and rural dwellers in mixed systems and is not expected to lead to CHE.

There was lack of insurance or other prepayment schemes that that would have mitigated the high level of CHE that was found. Approximately 99% of payments for healthcare by consumers were made using OOPS. However, protecting households from high OOPS is an important health system goal [13]. Protection against catastrophic health expenditures should be a priority item on the healthcare financing agenda [9]. Several international campaigns have advocated the removal of user fees, especially for primary health services [2], [32].

One limitation of the study is that we used a high threshold for catastrophic expenditures. If we had used a lower threshold, the levels observed would have been much higher. Other potential limitations include the use of cross sectional survey data with a recall period of one-month for outpatient care expenditures and six months for inpatient stays. In collecting data on household consumption of other goods and services using a one-month recall period, the accuracy could have been limited because expenditures on several items are incurred at different frequencies (daily, monthly, quarterly, and yearly) and may not be captured accurately in a one month period even if the expenditures are annualised. However, this appears to be the most feasible method. Using a rolling survey, so that part of the sample is interviewed at different time periods during the year, might address issues of seasonality.

Future studies should assess the real consequences for households of high levels of health expenditure and the thresholds that actually lead to catastrophe for different population groups. Such studies will require a qualitative and longitudinal design [33]. Future research should also determine the extent to which people who are enrolled in the NHIS and or other health insurance schemes are still exposed to OOPS and catastrophic expenditures of healthcare. A positive financial protective effect of health insurance, especially amongst the most-poor SES was found in Ghana [34]. Also, it was found that health insurance led to a four-fold decrease in incidence of CHE in Rwanda [35]. However, some authors noted that where the benefit package covered by the insurance is not comprehensive, households can still incur significant costs for medicines and outpatient visits [13].

All in all, the study found a lack of financial risk protection for healthcare in the study area and the worst affected were the rural dwellers and the poorest. OOPS, which was found to be regressive still dominates as the payment mechanisms for healthcare accounting for the very high level of catastrophic costs that were found in the study. Policymakers and programme managers in the two states should institute health reform mechanisms for developing, implementing and scaling-up financial risk protection mechanisms. The most-poor and rural dwellers should be particularly protected from incurring CHE.
